# A case of drug-induced parkinsonism and tardive akathisia with e1143g polymerase γ mutation-innocent bystander or a culprit?

**Published:** 2021-05-14

**Authors:** Pretty Sara Idiculla, Syed Taimour Hussain, Junaid Habib Siddiqui

**Affiliations:** ^1^University of Missouri Health Care, 1 Hospital Drive, Columbia, Missouri-65212, United States; ^2^United Medical and Dental College, Karachi, Pakistan

**Keywords:** atypical Parkinsonian disorders, polymerase γ 1-related Parkinsonism, polymerase γ mutation disorders

## Abstract

**Background and Aim:**

Polymerase γ (POLG) is a protein that plays a pivotal role in the replication of the mitochondrial genome. POLG-related disorders constitute a sequence of overlying phenotypes that can present from early infancy to late adulthood. Parkinsonism is the most common movement disorder associated with POLG mutation. We also summarize all reported cases of POLG-related Parkinsonism, along with a literature review.

**Case Description:**

We present the case of an 80-year-old male presented with complaints of episodic confusion, tremors, and restlessness. He has been on risperidone for psychosis. A normal DaT scan ruled out Parkinson’s disease, and molecular analysis for POLG was positive (E1143G). He was diagnosed with drug-induced Parkinsonism and tardive akathisia with an incidental POLG mutation.

**Conclusions:**

A literature search revealed 55 cases of “POLG-related Parkinsonism” that met our criteria. These mutations can clinically affect multiple organ systems. Parkinsonism is the most common movement disorder reported in association with POLG mutations. We conducted a molecular analysis in our patient due to his Parkinsonism and recurrent episodes of encephalopathy. E1143G mutation found in our case was coincidental and reported a non-pathogenic or benign variant in literature.

**Relevance for Patients:**

E1143G is a non-pathogenic variant and multiple studies have shown that its co-occurrence with other POLG mutations can aggravate disease occurrence and severity. Literature findings and the experience from our own case indicate that the pathogenicity of E1143G is debatable, and future studies involving this particular variant may help understand its pathological consequences.

## 1. Introduction

A group of proteins replicates the mitochondrial deoxyribonucleic acid (mtDNA), a major one being DNA polymerase γ (POLG). POLG-related disorders constitute a continuum of overlying phenotypes that can present from early infancy to late adulthood. The most common movement disorder found in association with POLG mutation is Parkinsonism. These patients usually show clinical symptoms at a young age and are commonly associated with progressive external ophthalmoplegia (PEO). We present an 80-year-old male with drug-induced Parkinsonism and tardive akathisia with an incidental POLG mutation. We performed a literature search to summarize all reported cases of POLG-related Parkinsonism.

## 2. Case Report

An 80-year-old Caucasian male with a past medical history of diabetes mellitus, chronic kidney disease stage III, obstructive sleep apnea, psoriatic arthritis on methotrexate, and depression on mirtazapine; presented with complaints of episodic confusion of 5 years duration, restlessness, and tremors of 2 years duration.

The episodic confusion was associated with apparent auditory/visual hallucination and delusions and typically occurred at night. The inciting event was a recognized infection during most episodes. He was started on risperidone to be used intermittently during these attacks to control the psychotic symptoms. According to his wife, he has taken risperidone only a few times after beginning the medication.

The restlessness comprised repeated crossing and uncrossing legs, sitting and standing, and moving his arms around. It began 3 years after initiating antipsychotic medications. Around the same time, he also developed tremors in his hands bilaterally (right >left). It has remained stable, is present predominantly at rest, and does not affect his day-to-day activities. He denied any loss of sense of smell, change in facial expression, voice change, drooling, constipation, and orthostatic symptoms. He also reported no similar complaints among his family members.

On examination, he had symmetric, akinetic, rigid Parkinsonism, and prominent akathisia. His total Movement Disorders Society Unified Parkinson’s Disease Rating Scale score was 46/104. Sensory examination showed impaired vibration sense in bilateral lower limbs. We advised him to avoid risperidone, as this could explain his Parkinsonism. When there was no noticeable improvement, we started him on a trial of Sinemet. A DaT scan was normal, ruling out Parkinson’s disease. We prompted genetic testing due to the occurrence of marked encephalopathy from minor infections and Parkinsonism from low and intermittent doses of risperidone. Molecular analysis was positive for a heterozygous POLG mutation- c.3428 A >G, p. Glu1143Gly (E1143G).

At 85 years of age, follow-up demonstrated a good response to Sinemet. The patient denied any new delirium episodes and mentions his tremors and akathisia is still present but improving. He was diagnosed with drug-induced Parkinsonism and tardive akathisia, with an incidental POLG mutation and peripheral neuropathy, most likely due to poor diabetic control.

Although E1143G mutation is a non-pathogenic POLG variant (https://www.ncbi.nlm.nih.gov/clinvar/variation/VCV000021312.5), it could be a contributing factor to our patient’s clinical presentation.

## 3. Literature Review

A literature search was done on PubMed using the keyword “POLG-related Parkinsonism.” We included all reported cases in the English language in our review and excluded those without the relevant documentation. A total of 55 patients met our criteria.

Delgado-Alvarado *et al*. summarized 37 cases of POLG-elated Parkinsonism from the year 2004 to 2014 [1]. Since then, 18 more cases were reported [2-11]. The characteristics of 19 patients, including ours, are summarized in Table 1 (2014–2020).

**Table 1:** Reported cases of POLG mutation Parkinsonism (2014-2020)

	Case	Age of onset (years)	Gender	Gene testing- Mutation	Onset of Park (years)	PEO	Myo	Neu	Other features	DaT scan	DA med
1	Miguel *et al*. [2]	56	Male	P648R W585X	56	+	-	+	• Ptosis • Dysphagia • Dysarthria	+	+
2	Miguel *et al*. [2]	46	Female	T251I P587L W585X	60	+	+	+	• Ptosis • Dysphagia • Facial diparesis • Exercise intolerance	+	+
3	Miguel *et al*. [2]	39	Make	P648R R807C	49	+	+	+	• Ptosis • Dysphagia • Dysarthria	+	+
4	Martikainen *et al*. [3]	50s	Male	A467T	72	+	-	-	• Short-term memory problems • Deceased	+	+
5	Martikainen *et al*. [3]	40s	Male	T955C	-	+	+	+	• Cerebellar ataxia • SNHL	+	+
6	Martikainen *et al*. [3]	50s	Female	T251I P587L	67	+	+	+	• RLS • Fatigue	+	+
7	Rempe *et al*. [4]	27	Female	G737R R853W	32	-	+	+	• Mild ptosis • Anxiety	+	+
8	Mehta *et al*. [5]	18	Male	E856K	18	-	-	-	• Dysarthria • Non-ambulatory • Cognitive difficulties • Dysphagia • Deceased	-	+
9	Mehta *et al*. [5]	19	Female	E856K	19	-	-	+	• Dysarthria • Ataxia	-	+
10	Kuo *et al*. [6]	30	Male	Y955C	48	+	-	+	• Ptosis • Ataxia • Slurred speech • Dysphagia	+	+
11	Schreglmann *et al*. [7]	50	Male	-	50	+	-	-	• SNP • Inattention • Pyramidal signs • Insomnia • Panic attacks • RLS • Neurogenic urge incontinence • Color blindness for blue	-	+
12	Schreglmann *et al*. [7]	63	Male	-	68	+	-	-	• Ptosis • Dysphagia	-	+
13	Schreglmann *et al*. [7]	61	Female	-	68	-	-	+	• Partial epileptic seizures • Vestibulocochlear dysfunction • Retinopathy • Psychosis • Stroke-like episodes	-	-
14	Meira *et al.* [8]	72	Male	T252I P587L R807C	72	+	-	+	• Ataxia	-	-
15	Hsieh *et al*. [9]	39	Male	R964C	39	-	-	-	• RBD • Additional GBA L444P mutation	-	+
16	Hsieh *et al*. [9]	70	Male	R964C	70	-	-	-	• Insomnia • Additional GBA L444P mutation	-	+
17	Ma *et al*. [10]	16	Female	S998L I898T	16	+	+	-	• Exercise intolerance • Dysarthria • Dysphagia • Ptosis • Optic atrophy	+	+
18	Chumarina *et al*. [11]	17	Female	G811A	24	-	+	-	• Early onset cataract • Premature ovarian failure	+	+
19	Current case	80	Male	E1143G	-	-	-	+	• Drug-induced Parkinsonism • Tardive akathisia • Recurrent encephalopathy episodes	Normal	+

*Park:* Parkinsonism; *PEO*: Progressive External Ophthalmoplegia; *Myo*: Myopathy; *Neu*: Neuropathy*; DA med*: Dopaminergic medication response SNHL: Sensory neural hearing loss; RLS: Restless legs syndrome; SNP: Supranuclear gaze palsy; GBA: Glucocerebrosidase gene; RBD: Rapid eye movement sleep behavior disorder; OSA: Obstructive Sleep Apnea; (+): Positive; (-): Not available/performed

- The age of onset of clinical symptoms ranged from 10 to 80 years, with an average onset between the 3^rd^ and 4^th^ decade.
- Among the 56 cases, 30 (53.6%) were males, 25 (44.6%) were females, and 1 patient had no gender specified.
- The age of onset of Parkinsonism ranged between 18 and 78 years- 4 patients (8.5%) had the onset before 20 years, 10 patients (21.3%) had between 21 and 40 years, 21 patients (44.7%) had between 41 and 60 years, and 12 patients (25.5%) had the onset after 60 years of age. PEO was a common manifestation in most cases, except 13 patients (23.2%). Myopathy was present among 28 patients (50%) and neuropathy among 30 patients (53.6%).
- Among the 25 patients who underwent DaT scan, 16 patients (64%) were positive and 43 patients (76.8%) showed good response to treatment with dopaminergic medications.

## 4. Discussion

The mitochondria are known as the cell’s powerhouse, generating adenosine triphosphate. Through the electron transport chain, this allows the proper functioning of cells, including the neurons. Multiple proteins that make up the system are derived from the mitochondrial genome, consisting of circular mtDNA.

POLG is a major DNA-binding protein involved in mDNA replication. POLG-related disorders can present with clinically overlapping symptoms in affected individuals. However, they are characterized into six clinical forms-

- Alpers-Huttenlocher syndrome- severe progressive encephalopathy, intractable epilepsy, and liver failure.
- Myocerebrohepatopathy spectrum- developmental delay, lactic acidosis, myopathy, and hepatic impairment.
- Myoclonic epilepsy myopathy sensory ataxia- a spectrum of epilepsy, myopathy, ataxia, and disorders previously described as a spinocerebellar ataxia with epilepsy.
- Ataxia neuropathy spectrum- sensory ataxia, neuropathy, dysarthria, ophthalmoplegia, and mitochondrial recessive ataxia syndrome.
- Autosomal recessive PEO- progressive extraocular muscle weakness causing ptosis and ophthalmoparesis without any systemic involvement.
- Autosomal dominant PEO- generalized myopathy, sensorineural hearing loss, axonal neuropathy, depression, Parkinsonism, hypogonadism, and cataracts.

Clinical signs and symptoms associated with POLG mutations include hypotonia, developmental delay, myoclonus, focal motor seizures, generalized seizures, status epilepticus, migraine, stroke-like episodes, Parkinsonism, chorea, sensory neuronopathy, ganglionopathy, axonal sensorimotor neuropathy, cerebellar ataxia, depression, psychosis, dementia, sensorineural hearing loss, PEO/ptosis, cataracts, retinopathy, liver failure, gastrointestinal dysmotility, proximal myopathy, exercise intolerance, diabetes mellitus, primary ovarian failure, ovarian dysgenesis, primary testicular failure, and cardiomyopathy [12].

Parkinsonism is the most common movement disorder associated with POLG mutations. The exact mechanism by which mitochondrial dysfunction leads to Parkinsonism is still not well understood. The existing hypothesis indicates that an alteration in the mtDNA can result in impaired protein synthesis resulting in mitochondrial dysfunction in the dopaminergic neurons [13]. Parkinsonism has been associated with both autosomal dominant and recessive POLG mutations and has an early age of onset, typically around the 3^rd^–4^th^ decade [14]. Although patients with POLG-related Parkinsonism have associated PEO, studies have reported its occurrence without PEO [9]. Tzoulis *et al*. described 11 patients with POLG-related encephalopathy. The patients had no signs of Parkinsonism; however, DaT scans indicated severe nigrostriatal neuronal loss [15]. Degeneration of the nigrostriatal dopaminergic pigment neurons is evident on DaT scans, and most cases respond to treatment with dopaminergic medications.

In 2004, Luoma *et al*. studied seven families, and for the 1^st^ time, described a significant link between Parkinsonism and POLG mutations [16]. The extent to which POLG variants play a role in the development of Parkinson’s disease is still not established. Studies of patients with idiopathic Parkinson’s disease have shown an increase in mtDNA deletions and impairment of oxidative phosphorylation in nigrostriatal neurons [17]. Luoma *et al*. also conducted a post-mortem examination on two patients with POLG mutation. This revealed loss of pigmented dopaminergic neurons in the substantia nigra, though Lewy bodies were not seen [16]. Betts-Henderson *et al*. performed an autopsy on a patient with multiple heterozygous POLG mutations. Microscopic examination of the brain showed a severe neuronal loss in the substantia nigra and Lewy bodies’ presence in the remaining nigral neurons [18].

E1143G mutation is a non-pathogenic variant found at varying frequencies in different population subgroups (https://www.ncbi.nlm.nih.gov/snp/rs2307441#frequency%20tab). The mutation is rendered benign due to its occurrence outside the identified pathogenic cluster [19]. Sequence analysis of the E1143G variant by computer-based algorithms has yielded mixed results. While one indicated a benign mutation (http://www.snps3d.org/), another program predicted it to be damaging protein structure and function (http://genetics.bwh.harvard.edu/pph/). The commonly encountered pathogenic POLG variants include A467T, W748S, G848S, and T251I-P587L [20]. Studies have shown that the co-occurrence of E1143G with other POLG variants may aggravate the disease’s occurrence and clinical severity. Horvath *et al*. reported five patients with E1143G mutation, of which two individuals had compound mutations- A467T/E1143G and S433C/E1143G [21]. Chan *et al*. investigated the biochemical consequences of POLG proteins with E1143G polymorphism. They described that the occurrence of W748S in cis with E1143 mutation could modulate the former’s phenotypic effects [22].

Our patient was positive for a heterozygous POLG mutation- c.3428 A >G, p. Glu1143Gly (E1143G). The variant probably remained silent till the dopamine blocking effects of risperidone were in effect. It could also be contributing to his episodic encephalopathy and amplifying his drug-induced Parkinsonism.

## 5. Conclusion

The mutation identified in our patient, a benign heterozygous POLG variant (E1143G), was incidental. The pathogenicity of E1143G is debatable, and future studies involving this particular variant may help understand the pathological consequences when it occurs as single or compound mutations.

### Conflicts of interest

The authors declare that they have no competing interests.
